# Multilayered blow-spun vascular prostheses with luminal surfaces in Nano/Micro range: the influence on endothelial cell and platelet adhesion

**DOI:** 10.1186/s13036-023-00337-9

**Published:** 2023-03-13

**Authors:** Iwona Łopianiak, Wiktoria Rzempołuch, Mehtap Civelek, Iwona Cicha, Tomasz Ciach, Beata A. Butruk-Raszeja

**Affiliations:** 1 Faculty of Chemical and Process Engineering, Warsaw University of Technology, Waryńskiego 1, 00-645 Warsaw, Poland; 2 Doctoral School of Warsaw University of Technology, Warsaw University of Technology, Pl. Politechniki 1, 00-661 Warsaw, Poland; 3 Section of Experimental Oncology Und Nanomedicine (SEON), Else Kröner-Fresenius-Stiftung-Professorship, ENT-Department, Universitätsklinikum, Erlangen, Germany; 4 Centre for Advanced Materials and Technologies CEZAMAT, Warsaw University of Technology, Poleczki 19, 02-822 Warsaw, Poland

**Keywords:** Multilayered small-diameter vascular grafts, Hemocompatibility, Endothelial cells, Solution blow spinning, Nanofibers, Magnetic seeding

## Abstract

**Background:**

In this study, two types of polyurethane-based cylindrical multilayered grafts with internal diameters ≤ 6 mm were produced by the solution blow spinning (SBS) method. The main aim was to create layered-wall prostheses differing in their luminal surface morphology. Changing the SBS process parameters, i.e. working distance, rotational speed, volume, and concentration of the polymer solution allowed to obtain structures with the required morphologies. The first type of prostheses, termed Nano, possessed nanofibrous luminal surface, and the second type, Micro, presented morphologically diverse luminal surface, with both solid and microfibrous areas.

**Results:**

The results of mechanical tests confirmed that designed prostheses had high flexibility (Young’s modulus value of about 2.5 MPa) and good tensile strength (maximum axial load value of about 60 N), which meet the requirements for vascular prostheses. The influence of the luminal surface morphology on platelet adhesion and the attachment of endothelial cells was investigated. Both surfaces did not cause hemolysis in contact with blood, the percentage of platelet-occupied area for Nano and Micro surfaces was comparable to reference polytetrafluoroethylene (PTFE) surface. However, the change in morphology of surface-adhered platelets between Nano and Micro surfaces was visible, which might suggest differences in their activation level. Endothelial coverage after 1, 3, and 7 days of culture on flat samples (2D model) was higher on Nano prostheses as compared with Micro scaffolds. However, this effect was not seen in 3D culture, where cylindrical prostheses were colonized using magnetic seeding method.

**Conclusions:**

We conclude the produced scaffolds meet the material and mechanical requirements for vascular prostheses. However, changing the morphology without changing the chemical modification of the luminal surface is not sufficient to achieve the appropriate effectiveness of endothelialization in the 3D model.

**Supplementary Information:**

The online version contains supplementary material available at 10.1186/s13036-023-00337-9.

## Introduction

Cardiovascular diseases (CVD) were responsible for 32% of all deaths worldwide in 2019 [1]. In advanced stages of CVD, the only choice is the surgical intervention, in which damaged arteries are replaced with autologous vessels or synthetic prostheses. However, the clinical success of this procedure is limited by low availability of autologous blood vessels. Also, commercially available synthetic grafts made of expanded polytetrafluoroethylene (ePTFE) or polyethylene terephthalate (PET) with diameters ≤ 6 mm frequently fail. The poor patency rate of synthetic prostheses [2–5] compels researchers to look for new approaches and solutions.

Although intimal hyperplasia or inflammatory complications may negatively affect the patency of the artificial vessels upon implantation, the main reason for prosthetic graft failure is occlusion caused by thrombosis [2–5]. In physiological conditions, the lumen of the blood vessel is covered with endothelial cells (ECs), which actively counteract the processes of platelet aggregation and blood coagulation through the synthesis and secretion of various bioactive substances e.g.: nitric oxide, heparan sulphate, prostacyclin [6]. In addition, intact endothelial monolayer inhibits the proliferation of smooth muscle cells (SMCs), limiting the risk of intimal hyperplasia. Implantation of synthetic grafts without this endothelium barrier may lead to surface protein adsorption followed by platelet adhesion, activation, and aggregation. Several strategies have been proposed towards quick endothelialization of prosthesis’ luminal surface. One of them is in vitro endothelialization, i.e. colonization of the prosthesis with the patient's cells before the implantation procedure. Another approach, in situ endothelialization, is based on colonization with ECs in the patient's body, which is possible through transanastomotic growth, transmural infiltration, and endothelialization with endothelial progenitor cells circulating in bloodstream [7].

Regardless of the approach chosen, the surface of the prosthesis must enhance the adhesion and proliferation of ECs, to enable restoration of a functional endothelium and, as a result, to reduce clotting processes. The literature proposes various strategies to improve EC attachment. One of them is based on the modulation of the surface topography e.g. by adding nanostructures to the lumen surface. This strategy assumes that introduction of nanostructures, e.g. nanofibres, increases surface to volume ratio and provides more binding sites for cell adhesion and biomolecule adsorption [8].

The ideal small-diameter vascular grafts (with diameter ≤ 6 mm) should mimic the layered structure of the native blood vessels and exhibit comparable mechanical properties. This leads to the idea of a layered prosthesis, where the inner surface is designed to provide an environment and topography suitable for reconstructing the endothelial layer, whereas the outer layers are tailored to fulfill other, specific purposes, i.e. ensuring appropriate mechanical properties and suitable porosity to enable the ingrowth of capillaries. To date, electrospinning has been the most universal and popular method of manufacturing fibrous vascular prostheses [9]. This technique enables the production of prostheses containing both micro- and nanofibers, as well as layered prostheses containing fibers of various sizes [10]. Nonetheless, electrospinning has a number of limitations related to high voltage requirements, low production rate, and limited number of suitable solvents [11]. Our group has developed an alternative technique for fabrication of fibrous vascular prosthesis, namely the solution blow spinning (SBS) method [12, 13]. The SBS system is similar to the electrospinning system but does not require the presence of  an electric field. The driving force of the process is the pressure of the working gas, which is fed to the nozzle together with polymer solution. The pressure forms fibers at the outlet of the nozzle and deposit them on the rotating collector. SBS has several advantages over electrospinning, including low cost, easiness to scale and control of the parameters, as well as no need for high voltage [14, 15].

Vascular grafts can be made of natural or synthetic polymers. Of the synthetic materials, PET or ePTFE were originally used. These materials are still the most commonly used in clinical practice for peripheral vessel replacement, but they have some disadvantages. Most importantly, their surface does not promote cell adhesion and they are quite resistant to chemical modifications. This is a significant disadvantage because the majority of synthetic polymers require surface modification in order to improve their biological properties. PUs are a chemically diverse group of polymers that are often studied in the context of biomedical applications. Heart valve, cartilage, skin, blood vessel and bone scaffolds have been successfully produced from PUs [16–23]. The versatility of PU-based scaffolds arises from material bio- and hemocompatibility, its easy processing, and appropriate mechanical properties [24]. Furthermore, the mechanical properties of PUs, including elasticity, strength, hardness, and resiliency, are easily controllable by changing the ratio of soft and hard segments. [25, 26]. Moreover, PUs present attractive biological properties probably due to the fact that urethane bond is similar to the peptide bond. Cells, including ECs, are able to adhere to the PU surface, even without the application of chemical modifications. This makes it possible to study the influence of the topography (fiber diameter etc.) on the cell-surface interactions. In this study, medical grade ChronoFlex PU was selected because of its reportedly high athrombogenicity.

In our previous study, we evaluated the influence of fibrous surface morphology on endothelial and smooth muscle cell (SMC) growth [27]. We have shown that both morphology (solid versus fibrous) and average fiber diameter (submicron fibers versus microfibers) of scaffolds influenced the growth of ECs. Here, we designed layered cylindrical prostheses that differ in the morphology of the luminal surface. The aim of the present work was to compare two types of prostheses with multilayered walls. The outer layer is made of aligned microfibers, with an average diameter of about 1000 µm, which are intended to support the SMCs development. The middle layer with total layer thickness of about 500 µm, containing non-aligned microfibers with an average diameter of about 1000 µm is expected to give the prosthesis adequate flexibility and mechanical strength. Finally, the internal layer is composed of dense microfibers presenting with two morphological types of luminal surface. This layer is designed to support the attachment of ECs by ensuring the appropriate topography, either a mixed solid/microfiber structure or a nanofiber structure. The prosthesis termed “Micro” has a luminal surface composed of solid areas (flat, film-like surfaces without fibrous structures) and microfibers, while in the prosthesis termed “Nano” the luminal surface is composed of nanofibers. Following the fabrication of the prostheses, their physical properties were characterized. Further, hemocompatibility of the distinct luminal morphologies was compared using human platelets, and two cell seeding models were used to evaluate the growth of ECs on Nano versus Micro surfaces.

## Materials and methods

### Vascular prostheses fabrication

Prostheses were produced from medical grade polyurethane solution by SBS method, as described elsewhere [12, 27]. Briefly, polyurethane ChronoFlex®C75A (Advanced Biomaterials, USA) was dissolved overnight in 1,1,1,3,3,3-hexafluoro-2-propanol (> 99%Fluorochem Ltd, UK) on magnetic stirrer. The polymer solution was transferred into syringe and fed to the inner nozzle of concentric nozzle system. The polymer solution flow rate was controlled by syringe pump. The inner diameters of inner and outer nozzles were 1.1 and 10 mm, respectively. Fibers were collected on rotating collector, 6 mm in diameter and 12 cm in length, mounted 10–30 cm away from the tip of inner nozzle. Prior to the SBS process, the collector was covered with a thin layer of 10% w/v poly(ethylene) glycol 2000 (Sigma Aldrich, Germany) solution in distilled water in order to simplify removal of the prosthesis from the collector surface. After the prosthesis deposition and its immersion (together with the collector) in distilled water for 2 min, the prosthesis was gently slid off the collector. The slight shrinking of the prostheses after the removal resulted in a final inner diameter of 5 mm.

**Fig. 1:**
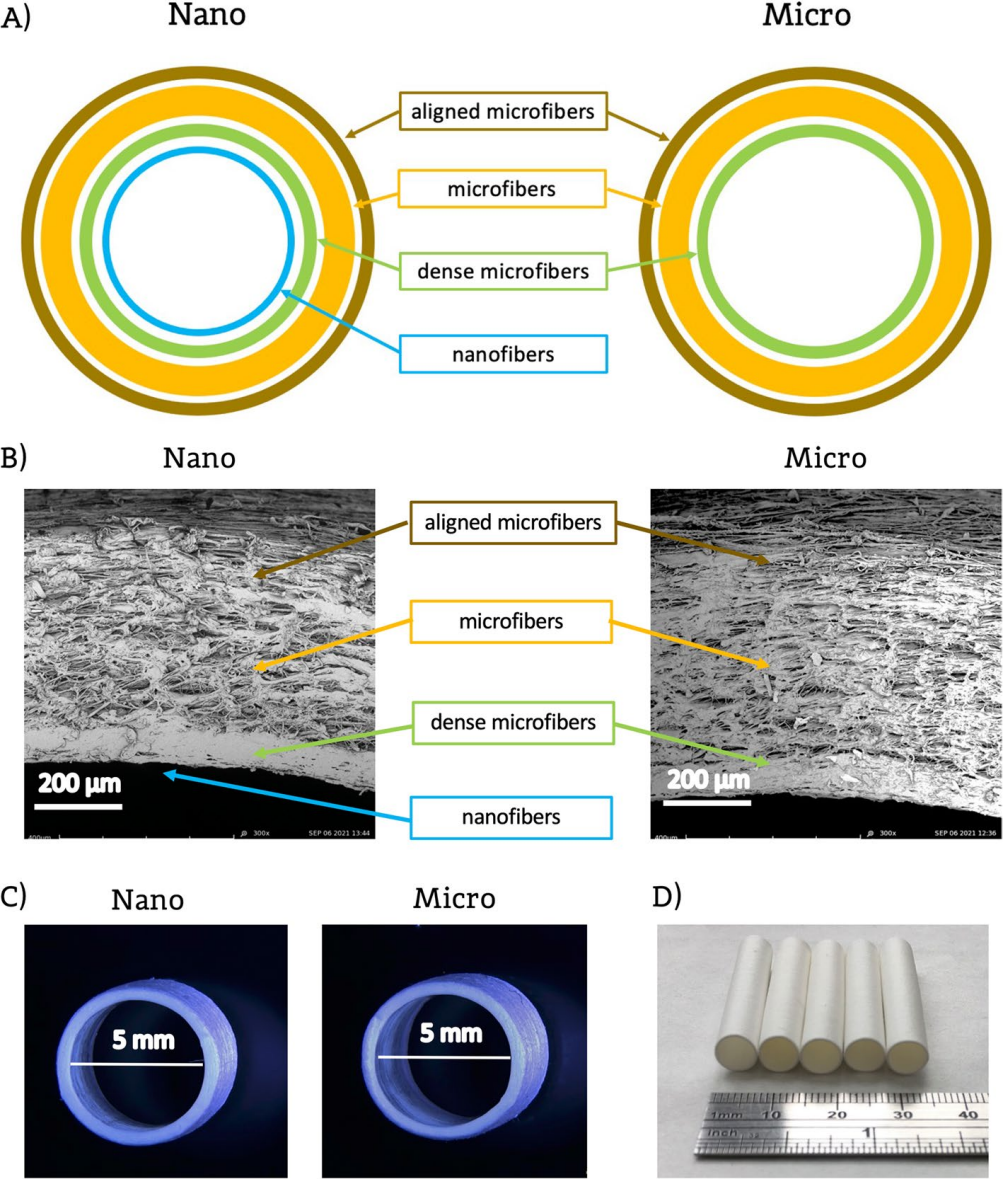
**A** Layers arrangement in Nano and Micro prosthesis, (**B**) Cross section of Nano and Micro prosthesis’ wall, (**C**) stereoscopic image of Nano and Micro prosthesis, (**D**) macroscopic image of prostheses (Nano and Micro mix)

Two variants of layered prostheses were produced: (a) Nano and (b) Micro. As shown in Fig. 1A, Nano prosthesis consists of the following layers: nanofibers (luminal), dense microfibers, microfibers, and aligned microfibers (outermost). Micro prosthesis consists of the following layers: dense microfibers (luminal), microfibers, and aligned microfibers (outermost). The SBS process parameters used for producing individual layers are shown in Table 1.

**Table 1:** SBS process parameters applied for each layer in Nano and Micro prostheses. The layer that is present in a given prosthesis type is marked with “ + ”, a layer that is absent is marked with “- “

Layer	Nano	Micro	Polymer conc. [%_w/w_]	Solution vol. [ml]	Collector-nozzle tip distance [cm]	Collector rotational speed [rpm]	Solution flow rate [ml/h]	Air flow rate [MPa]
nanofibers	+	-	2	3	50	3 000	30	0.1
dense microfibers	+	+	5	6	10	3 000	30	0.1
microfibers	+	+	5	20	30	3 000	30	0.1
aligned microfibers	+	+	5	4	30	20 000	30	0.1

### Morphology of the prostheses

The prostheses were cut open and flat samples with dimensions 0.5 × 0.5 cm were glued to the SEM stubs with conductive carbon adhesive tape. Samples of internal (*n *= 3) and external surfaces (*n* = 3) were prepared. To characterize cross-sectional sample’s morphology, samples of prostheses 0.5 cm in length (*n* = 3 for each type) were glued upright to SEM stubs. The samples were then coated with 15 nm of gold using sputter coater (K550 Emitech, Quorum Technologies). Images of every sample (*n* = 10) were taken at magnifications × 200, × 600, and × 5000 using scanning electron microscopy Phenom G1 (Phenom World). SEM images were used to determine fiber diameter, pore size, and prostheses thickness. To determine the percentage of fibrous area on the internal luminal surface of Micro prostheses, the percentage of fibrous surface was measured in *n* = 20 SEM images. In every sample, *n *= 100 fiber diameters were measured using Fiji software. For nanofibrous internal surface of Nano prostheses, pore size was determined using SEM images of luminal surface at magnification × 5000. For this, the threshold tool (Fiji software) was used to delineate the most surface pores and the area of *n* = 100 pores was measured using Fiji software The pores were approximated to be circular in shape and the pore size (diameter) was determined using the circle area formula.

3D view of cylindrical structures was provided by a stereoscopic microscope Leica M205 C (Leica Microsystems GmbH).

Porosity was determined individually for every prosthesis. Two prosthesis ends, 1 cm in length were cut off and weighted on analytical lab scale. Afterward, the samples (*n* = 2 for each prosthesis) were glued upright to SEM stubs and coated with 15 nm of gold as described above. SEM images (*n* = 6) at magnification 200 × were taken, and *n* = 30 wall thickness measurements were made for each sample to determine individual layers' thickness and total wall thickness. The results (sample weight (m_s_) and total wall thickness (${\updelta }_{\mathrm{s}})$) were averaged and used to determine prostheses porosity ($\upvarepsilon )$ using formula:$\upvarepsilon =\left(1-\frac{\frac{{\mathrm{m}}_{\mathrm{s}}}{{\updelta }_{\mathrm{s}}\bullet {\mathrm{S}}_{\mathrm{s}}}}{{\uprho }_{\mathrm{p}}}\right)*100\%$, where ${\uprho }_{\mathrm{p}}$ is a density of polyurethane ChronoFlex®C75A, ${\uprho }_{\mathrm{p}}=1.2\mathrm{ g}/{\mathrm{cm}}^{3}$ [28], ${\mathrm{S}}_{\mathrm{s}}$ is a sample’s side surface determined using formula ${\mathrm{S}}_{\mathrm{s}}=2\uppi (\mathrm{r}+\updelta )\mathrm{L}$, where r is a prosthesis inner radius, *r* = 0.25 cm and L is a sample length L = 1 cm. The results are presented as mean value ± SD.

### Mechanical properties

Prostheses of 5 cm in length (*n* = 5 for each type) were placed in the pneumatic jaws of the testing machine Instron 3345 equipped with 50 kN static load cell. Prostheses were stretched at the rate of 10 mm/min until the break. Dedicated Bluehill software automatically determined maximum load, elongation at break, Young’s modulus, and ultimate tensile stress. The results are presented as a mean value ± SD.

### Leakage and delamination tests

The leakage test was carried out as follows: prostheses of 4 cm length (*n* = 3 for each type) were mounted in a closed flow system connected to a peristaltic pump Zalipm PP1B-05A (Zalipm) and 0.9% NaCl solution was circulated in the system (through the prosthesis) for 1 h at a flow rate of 20 ml/min. During the test, samples were checked for any signs of leakage through the prostheses’ walls. After the leakage test, prostheses were dried at 20 °C for 24 h. Then, the samples were glued to SEM stubs with conductive carbon adhesive tape and covered with 15 nm layer of gold. Materials cross-sections were analyzed using scanning electron microscopy Phenom G1.

The above-described flow system was also used to test the permeability of the prostheses’ walls in contact with blood. Freshly drawn whole blood was connected to the flow system and the prostheses were perfused for 1 h at a flow rate of 20 ml/min. During that time, macroscopic observations were carried out to assess whether there is any blood leakage through the prosthesis’ wall.

Additionally, a static delamination test was carried out. Prostheses of 1.5 cm length (*n* = 3 for each type of prostheses and for each timepoint) were prepared and placed in 1.5 ml Eppendorf® test tubes fully filled with 0.9% NaCl solution. Test tubes were closed and placed in an incubator at 37 °C for 7, 14, or 30 days. After this time, the prostheses were dried at 20 °C for 24 h and investigated using scanning electron microscopy Phenom G1.

### Hemocompatibility of materials

Blood tests were performed using fresh human blood from healthy volunteers. Blood was collected in 1.8 ml test tubes containing citrate (BD Vacutainer, Franklin Lakes, NJ, USA).

### Static platelet adhesion

For static analysis, round shape samples (*n* = 2 for each type of material) were placed in 24-well plate with the luminal surface of the prosthesis facing up. In order to stabilize and flatten the material, each sample was placed in CellCrown (Sigma-Aldrich) inserts. Subsequently, 500 µl of 0.9% NaCl solution in ultrapure water was added to wells with samples and plate was incubated at 37 °C for 30 min. Then, NaCl solution was removed and 200 µl of platelet-rich plasma (PRP) was added to every well containing the samples. PRP was prepared using two “slow” centrifugations: 150 g for 14 min (first centrifugation) and 150 g for 12 min (second centrifugation). The platelet density in PRP was 1 × 10^6^ platelets/µL. Plate with materials was incubated at 37 °C for 90 min. Next, PRP was removed, and samples were thoroughly rinsed with 0.9% NaCl to remove blood residues. Finally, samples were prepared for SEM analysis. Briefly, materials were incubated in 4% paraformaldehyde for 24 h at 4 °C. Next, the samples were dehydrated by 10 min immersion steps in 50, 60, 70, 80, 90, and 100% ethanol solution (EtOH), and for 20 min in 1:2 hexamethyldisilazane:ethanol (HMDS:EtOH), 2:1 HDMS:EtOH and 100% HDMS solution. Finally, the samples were glued to SEM stubs with conductive carbon adhesive tape (luminal surface of prostheses up) and covered with 15 nm layer of gold. The % of platelet-coated area was counted from SEM images of every sample, taken at 3000 × magnification. Additionally, pictures at magn. = 5000 × were taken in order to present the morphology of surface-adhered platelets in detail. The platelet adhesion assay was done in triplicate, with change of blood donor each time. For every sample *n* = 10 SEM images were taken. The average values for all materials were calculated from 60 images (10 images × 3 experiments × 2 samples).

In this assay, PTFE was cut from vascular prosthesis (FlowLine Bipore, Jotec) and used as a reference material that induces low platelet adherence.

### Hemolysis

Round samples with diameter of 14 mm (*n* = 3 for each type of prosthesis) were placed in 48-well plate with the luminal surface of the prosthesis facing up. To separate erythrocytes from plasma, fresh blood was centrifuged at 700 g for 5 min and plasma was removed from blood tubes. Then, erythrocytes were diluted 20 × in ultracold PBS and 500 µl of erythrocyte suspension was added to wells with materials. PBS was used as a negative control and 0.2% TritonX-100 as a positive control. Triplicate samples were placed on a shaker at 300 rpm for 1 h, at 37 °C. Afterward, 600 µl of solution from every well was centrifuged at 700 g for 1 min, and 200 µl of supernatant was transferred triplicate to 96-well plate. The absorbance at 540 nm was measured using a plate reader Epoch Biotek (Biokom).

Hemolysis rate was calculated using the following formula:

$$HR=\frac{{A}_{S}-{A}_{CN}}{{A}_{CP}-{A}_{CN}}*100\%$$

where: A_S_ – sample absorbance, A_CP_ – mean positive control absorbance, C– mean negative control absorbance.

Results are presented as mean hemolysis rate ± SD.

### Endothelial cell culture

Human umbilical vein endothelial cells (HUVECs) were isolated from freshly collected umbilical cords (kindly provided by the Dept. of Gynaecology, University Hospital Erlangen) and grown in supplemented endothelial cell growth medium (EGM-2, Promo Cell, Germany). Accutase solution was used for cell harvesting. Cells from passages 1 or 2 were used in experiments. All experiments were repeated 3 times, in each experiment the material was used in duplicate. The use of human material was approved by the local ethics committee at the University Hospital Erlangen (review number 14-85_3-B from 01.02.2022).

### Static cell seeding on flat materials – 2D model

Flat samples were cut off from cylindrical grafts, sterilized with 70% ethanol, washed with sterile PBS, and placed in 24 well cell culture inserts. Then, materials were seeded with HUVECs (5 × 10^4^ cells/sample) and incubated at 37 °C for 1, 3, and 7 days. Culture media were changed 24 h after seeding and then every second day.

To analyze cell viability, Alamar Blue assay was performed according to manufacturer’s protocol. Briefly, after 1, 3, or 7 days of cell culture, materials with cells growing on the surface were transferred to a new 24-well plate and gently washed with sterile PBS. Then Alamar Blue working solution was added to each well (500 µl/well) and incubated with samples at 37 °C for 18 h in the dark. The fluorescence of the Alamar Blue solution was measured at Ex./Em = 550/590 nm using a plate reader (SpectraMax iD3, Molecular Devices).

### Magnetic cell seeding on cylindrical prostheses – 3D model

Cell seeding was also performed on cylindrical vascular prostheses. For this, all materials were cut to equal length of 5 cm. Samples were sterilized with 70% ethanol, washed with sterile PBS, and placed in transparent cell culture tubes. 1% agarose solution was used to fix the prostheses in a vertical position inside the cell culture tubes. Before cell seeding prostheses were preincubated with EGM-2 medium for at least 1 h.

HUVECs were seeded on the lumen of the prostheses using magnetic seeding technique. Cells were pre-incubated with superparamagnetic iron oxide nanoparticles (SPIONs) in cell culture flasks for 24 h at 37 °C as described before [29]. After incubation, the SPION-loaded cells were harvested and counted. HUVECs were suspended in the culture media and transferred into the luminal space of each prosthesis (1 × 10^6^ cells/prosthesis). Immediately after transferring the cell suspension, the scaffolds were exposed to a radially symmetric magnetic field for 15 min using the VascuZell endothelizer (Vascuzell Technologia S.L., Madrid, Spain). The cell culture tubes with prostheses were then carefully removed from the endothelizer and placed in the incubator for 1, 3, or 7 days. The culture medium was changed 24 h after seeding and then every second day.

### Cell staining and image analysis

After the given cell culture period, cells growing on the lumen surface were fixed with 4% buffered paraformaldehyde (Roth GmbH, Karlsruhe, Germany) and permeabilized with 0.2% Triton X-100 (Sigma-Aldrich, Munich, Germany) in PBS. F-actin filaments were stained by Alexa488-phalloidin (Invitrogen, Thermo Fisher) and visualized using fluorescence microscope Zeiss Axio Observer Z1 (Zeiss, Jena, Germany) at 10 × magnification. To observe cells growing inside cylindrical prostheses, the materials were cut along the longitudinal axis, pressed to the glass slides, and then visualized using multiple mode (2 × 5). Cell counting was performed using the ImageJ software (Fiji, version 1.47v).

### Data analysis and statistical analysis

#### 2D cell culture model and platelet adhesion assay

Cell coverage was calculated in 6 circular samples with a diameter of 11 mm (2 replicates × 3 independent experiments). For each sample, at least 3 microscopic images (magnification = 10x) were taken in randomly selected places and the cell coverage was calculated for every image. The average coverage was then calculated for each sample and the resulting boxplot was based on these 6 average values for all 6 samples. A boxplot in a%-b% range indicates that in a given group of materials, there was at least one sample with a% coverage and at least one sample with b% coverage.

#### 3D cell culture model

Cell coverage was calculated in 5 cylindrical samples (diameter 6 mm, length 5 cm) from 3 independent experiments). For each sample, at least 2 multi-tile scan microscopic images (magnification = 10x) were taken. Each “tile” represents the standard analysis area at 10 × and the multi-tile scans covered the surface of 2 tiles (prosthesis circumference) × 5 tiles (prosthesis length), corresponding to an area of approx. 1.3 mm × 4.5 mm. For each sample, 2 multi-tile scans were performed and the results were averaged. Based on 5 averaged values for all 5 samples a boxplot was plotted in a%-b% range, indicating that in a given group of materials, there was at least one sample with a% coverage and at least one sample with b% coverage.

The results of the other measurements (mechanical analysis, delamination assay, hemolysis) were presented as mean values ± SD. Statistical significance of differences was analyzed using single-factor or two-factor analysis of variance (ANOVA) for p < 0.05 with post-hoc Tukey’s test (OriginPRO 2020b).

## Results

### Morphology of prostheses

In the first part of this study, we produced and compared two types of vascular prostheses. The wall of prostheses consisted of several layers, each of which should fulfill specific functions. A schematic diagram comparing the arrangement of layers in the respective types of prostheses is shown in Fig. 1A, while Fig. 1B shows SEM pictures of their wall cross-sections. Essentially, the two scaffold types differed by the presence of nanofibrous layer on the luminal side of the Nano prosthesis. Our earlier studies have shown that prostheses made only of microfibers are leaky. Therefore, we decided to include a layer of densely arranged microfibers in both types of prostheses. This layer, whose thickness was about 10% of the total wall thickness acted as a sealing. The thickness of this layer was selected as a result of our previous work (data not shown), and the prostheses’ permeability was tested in a flow system using saline (see below). The next layer to the outside is a layer of loosely arranged microfibers, intended mainly to ensure appropriate mechanical properties of the prostheses (e.g., flexibility) and to achieve the desired wall thickness. This is the thickest layer of the graft, constituting about 80% of the total wall thickness. The thin outermost layer, representing about 10% of the total wall thickness consists of circumferentially aligned fibers and is designed to promote attachment of SMCs. Figure 1C and Fig. 1D show microscopic (stereoscopic microscopy) and macroscopic photos of prostheses. The macroscopic appearance of both types of prostheses was similar.

**Fig. 2:**
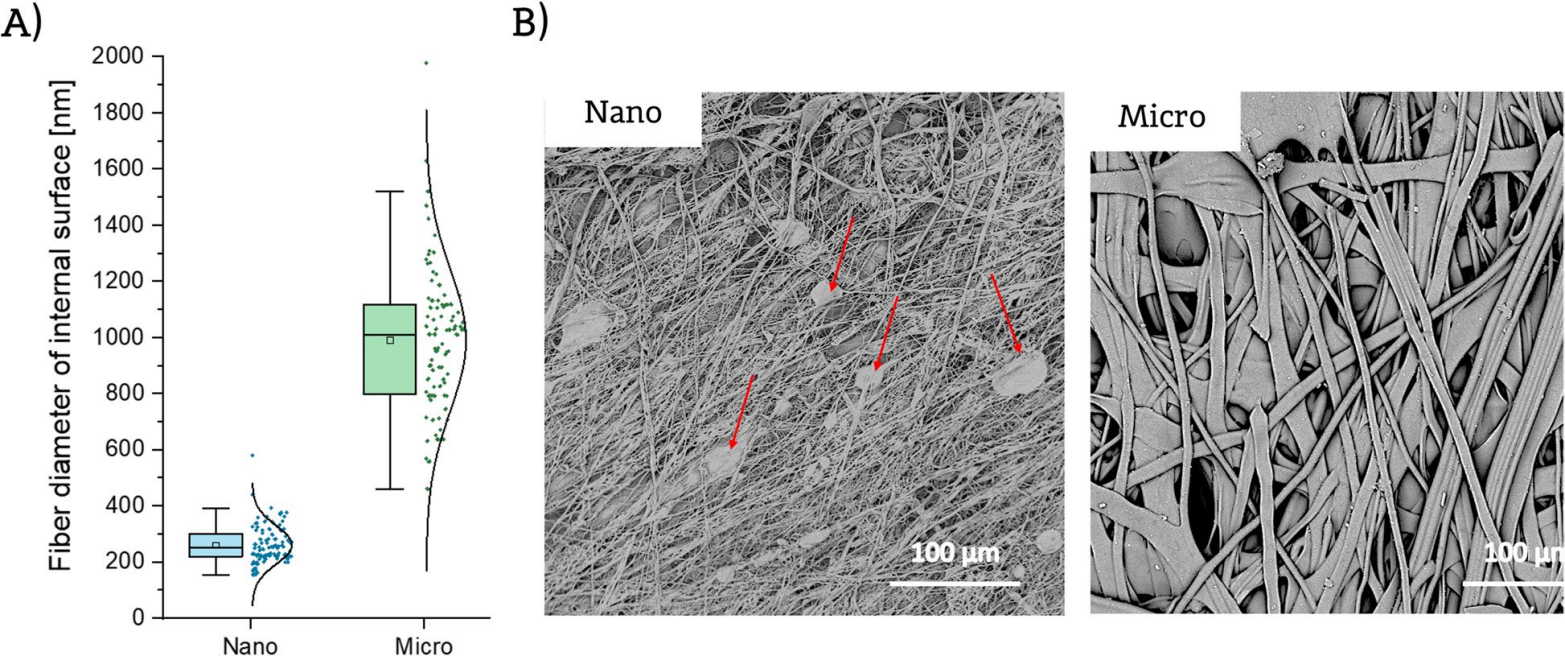
**A** Fiber diameter distribution and (**B**) internal surface morphology (arrows indicate defects present on the surface) for internal surface of Nano and Micro prostheses

The evaluation of fiber diameter on internal surfaces of both types of prostheses is presented in Fig. 2. Average fiber diameters of luminal surfaces of Nano and Micro prostheses were 262 ± 68 nm and 991 ± 251 nm, respectively. Additionally, pore size measurements were performed on the luminal surface of prostheses. Average size of pores was 2.5 ± 0.9 µm for Nano and 3.7 ± 1.7 µm for Micro scaffold. It must be noted that in Micro prosthesis, the fibrous areas covered only about 14% of luminal surface.

The described SBS process allowed to obtain prostheses with comparable properties, but with differing lumen topography. The luminal surface of Nano prostheses was characterized by nanofibers with single defects (beaded fibers) present on the surface (as indicated by the arrows in the Fig. 2B), while the luminal surface of Micro prostheses had a more heterogenous structure, including areas of smooth solid surface with pores and small fibrous areas with large, flattened fibers.

### Mechanical properties of prostheses

**Fig. 3:**
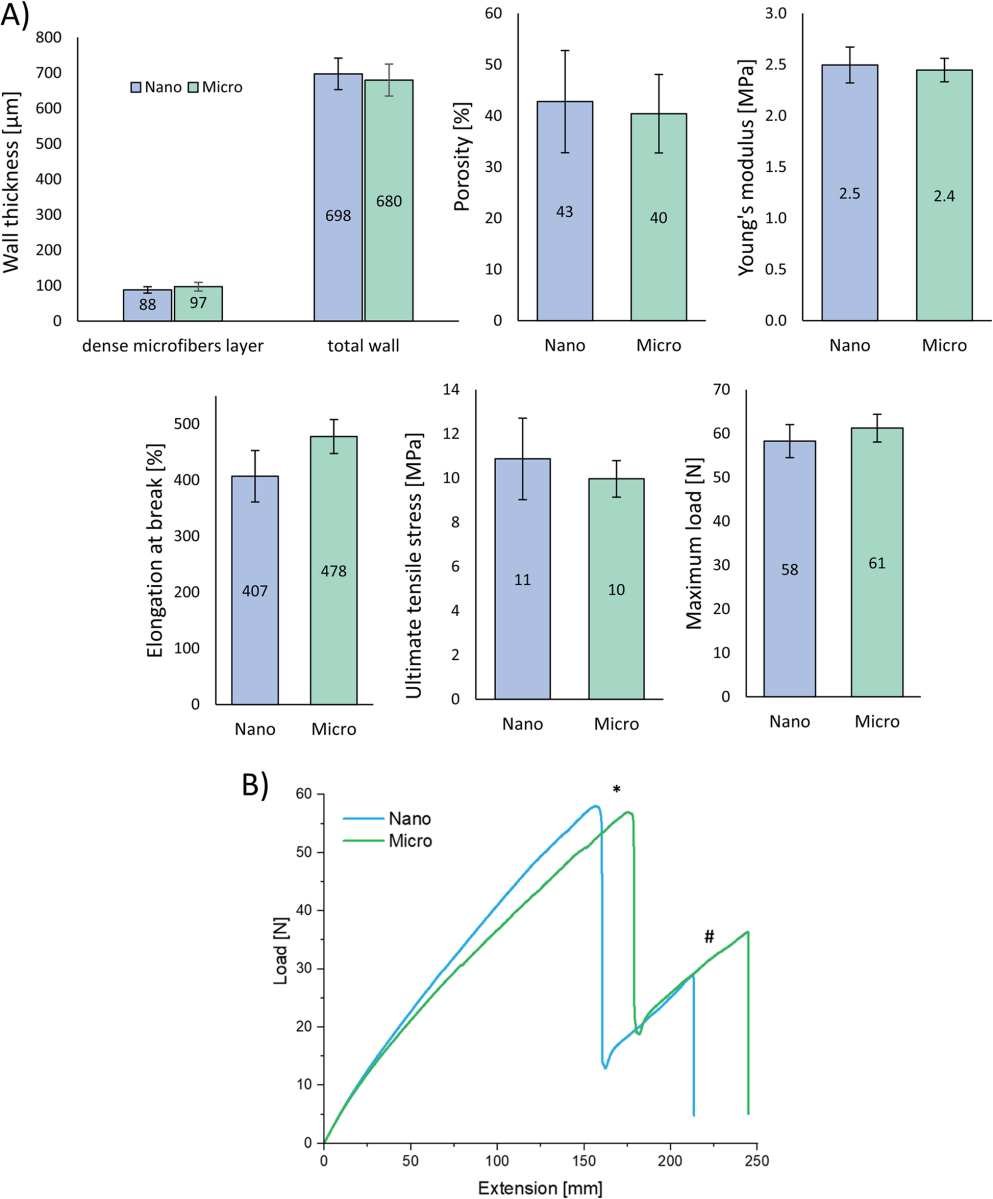
**A **Mechanical properties of Nano and Micro prosthesis (*n* = 5), (**B**) Load-extension curve for Nano and Micro prostheses. “*” indicates a change-point related to the rapture of the two outer microfiber layers, # indicates a change-point related to the rapture of the internal dense microfiber layer

As shown in Fig. 3A, the mechanical properties of Nano and Micro prostheses were similar. No significant differences regarding wall thickness were observed between the two types of prostheses. The total wall thickness was 698 ± 44 µm for Nano and 680 ± 45 µm for Micro scaffolds. The thickness of innermost nanolayer for Nano prostheses was 9 ± 2 µm, whereas the thickness of dense microfibers’ layer was 88 ± 9 µm for Nano and 97 ± 12 µm for Micro.

In accordance with this, no significant differences were detected in mechanical properties of the prostheses. Both types presented elastic behavior with high elongation at break values. Porosity was similar and equaled 43 ± 10% for Nano and 40 ± 8% for Micro. Young’s modulus values for Nano and Micro prostheses were 2.5 ± 0.2 MPa and 2.4 ± 0.1 MPa, respectively, while the respective maximum load values were 58.3 ± 3.8 N and 61.2 ± 3.2 N. Ultimate tensile stress for porous sample was 10.9 ± 1.8 MPa for Nano and 10.0 ± 0.8 MPa for Micro prostheses. Elongation at break value was lower for Nano (407 ± 46%) than for Micro prostheses (478 ± 30%), but the difference was not statistically significant. Figure 3B shows a typical load-extension curve. The shape of the curve is similar for both types of prostheses. The first change-point (marked as “*”) in the curve is related to the rupture of the two outer microfibrous (aligned and non-aligned) layers of the prosthesis. The test ended when the remaining layer (dense microfibers) was ripped up (the second change-point marked as “#”).

### Prosthesis leakage and delamination test

**Fig. 4:**
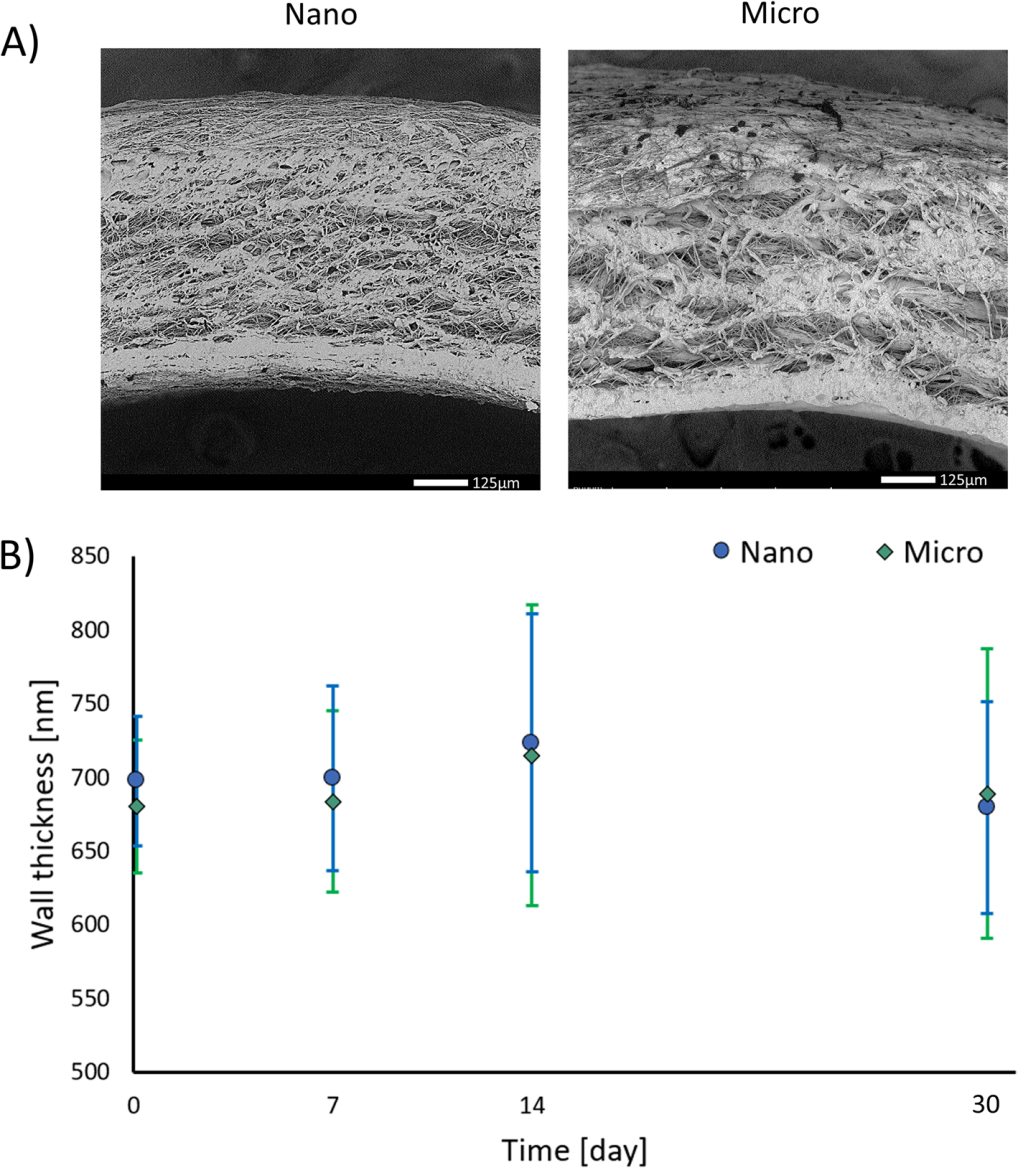
**A** Prostheses cross-section SEM images after 30 days of static delamination tests, (**B**) Prostheses wall thickness before and after static delamination test

During 1 h contact between Nano or Micro prostheses and 0.9% NaCl solution in flow system, no soaking or leakage was observed. No leakage was also observed during blood contact analysis in flow system. Additionally, no delamination of layers was observed either after 1 h contact with 0.9% NaCl solution in a flow system during dynamic delamination test, or after 30 days of incubation in 0.9% NaCl (static delamination test). Representative SEM images of Nano and Micro prostheses, showing their cross-sections after 30 days of static incubation in 0.9% NaCl solution, are presented in Fig. 4A. Cross-section SEM images of Nano and Micro prostheses after dynamic (1h) and static (7 and 14 days) analysis are presented in supplementary data. Figure 4B presents the results of wall thickness measurements before and after 7, 14, and 30 days of static delamination. No significant changes in wall thickness were observed, regardless of duration of the test and the type of prosthesis.

### Biological evaluation

The detailed characterization of produced scaffolds demonstrated that Nano and Micro prostheses differ only in their luminal surface morphology. In the second part of this study, we, therefore, evaluated the influence of this structural difference on hemocompatibility and endothelial cell attachment to the produced scaffolds.

### Platelet adhesion

**Fig. 5:**
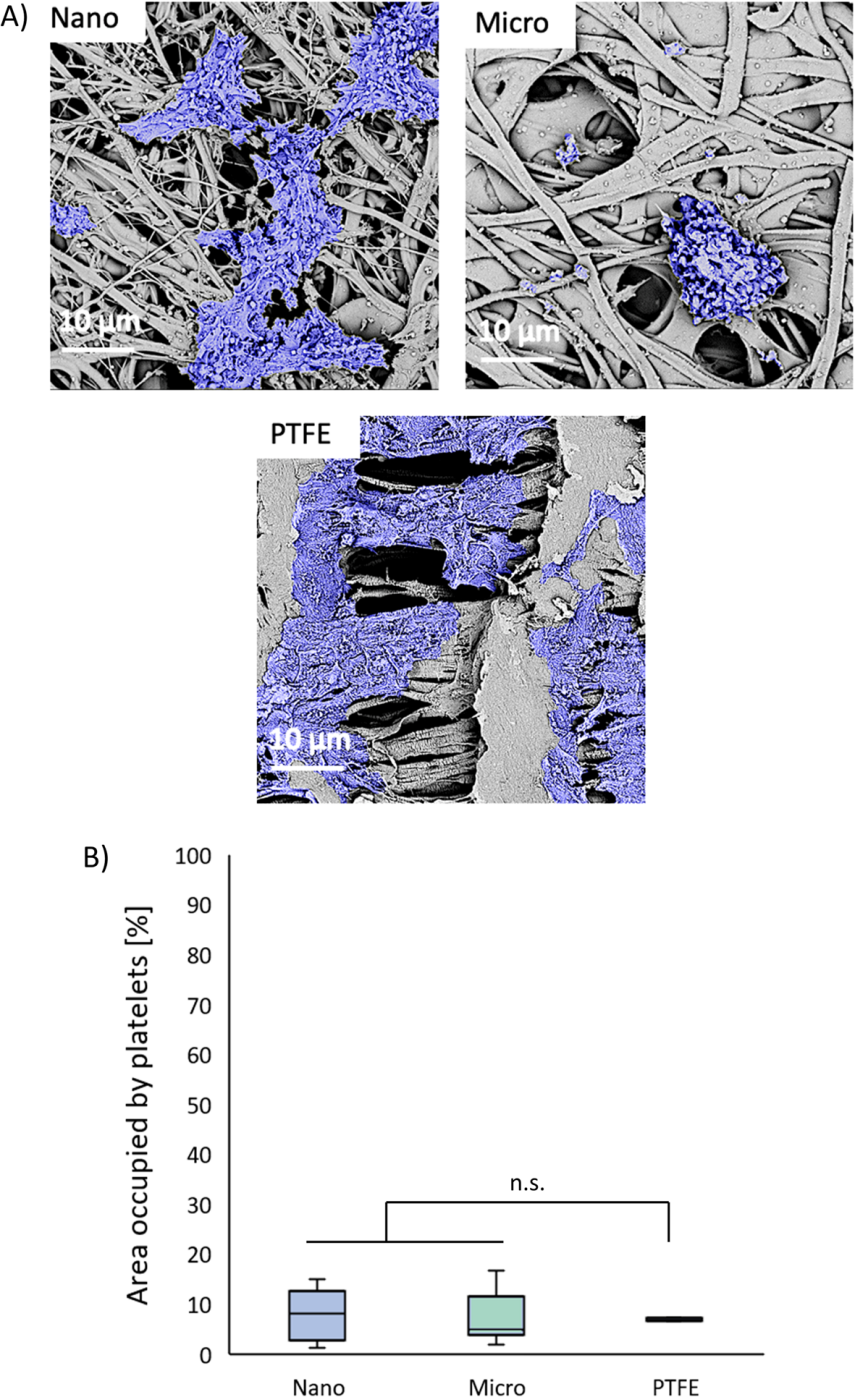
**A **Morphology of surface-adhered platelets (magn. = 5 kx) and (**B**) percentages (*n* = 6) of platelet-occupied area for Nano and Micro surfaces. PTFE was used as a low-thrombogenic reference material

The luminal surface of the materials after the platelet adhesion test is shown in Fig. 5A. The percentage of the platelet-occupied area is shown in Fig. 5B. The average values obtained for all tested materials were similar and no statistically significant differences were detected (p > 0.05 for all pairs). However, SEM images pointed to the differences in the morphology of the adherent platelets. On the Nano surfaces, platelet aggregates formed strongly flattened structures. There was a relatively large variation in platelet coverage between samples, ranging from 1 to 19% and the average value of platelet coverage was 8.6 %. In the case of Micro materials, a different morphology of the adhered platelets was observed. The cells formed relatively large aggregates, which had a spherical, rounded form. Highly flattened aggregates were rare. The variation in platelet coverage values between the samples was similar to Nano, in the range of 2–17% and the average value of platelet coverage was 6.2%. The average platelet coverage values for both types of prostheses were close to those observed on the surface of PTFE (7.0%). In the case of PTFE, the adherent platelets formed highly flattened layer and no spherical aggregates were observed.

### Hemolysis

The results presented in Table 2 demonstrated that hemolysis rate upon blood contact with Nano or Micro prostheses was < 1%. The produced prostheses thus do not cause blood hemolysis.

**Table 2:** Hemolysis rate of Nano and Micro prostheses

	Nano	Micro
Hemolysis rate [% of positive control]	0.4 ± 0.1	0.1 ± 0.1

### Endothelial cell culture

#### Static seeding on flat materials – 2D model

**Fig. 6:**
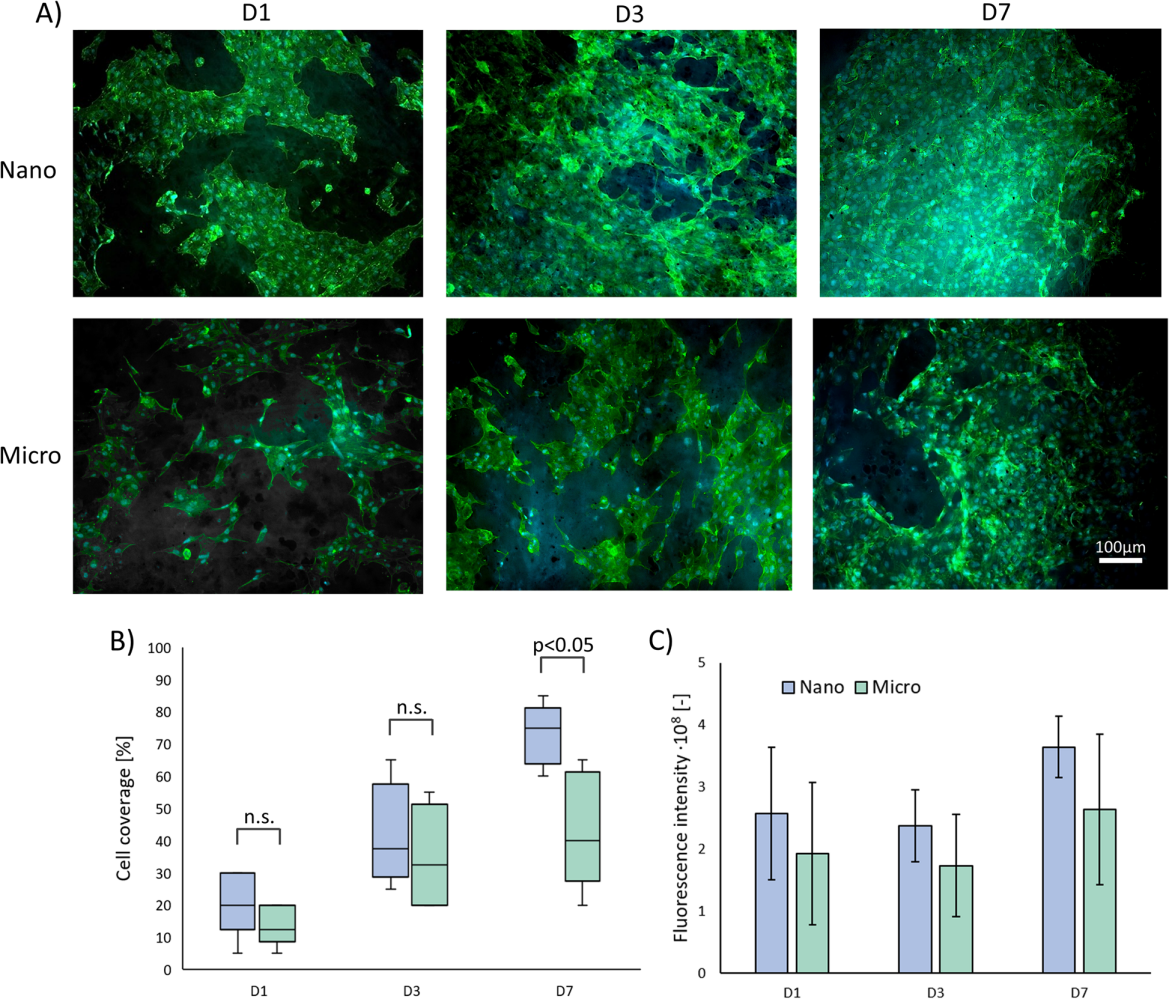
Cell culture with flat materials: (**A**) HUVECs growth, (**B**) cell coverage and (**C**) cell viability after 1,3 and 7 days of culture (*n* = 6)

The results of cell culture on flat samples (the 2D model) are shown in Fig. 6. Microscopic analysis showed that the cells showed the correct morphology and adhered to the surface of the fibers (Fig. 6A). Starting from the first day of culture (D1) a higher percentage of cell-covered area (Fig. 6B) was detected on Nano surfaces, but the differences were not statistically significant. Cellular coverage for the Nano surface was in the range of 5–30%, with an average value of 20%. For Micro surfaces, cellular coverage values ranged from 5 to 20%, with an average of 13%. A similar relationship was obtained on the third day of culture (D3). In the case of Nano surfaces, the cellular coverage values were higher and ranged from 25 to 65%, with an average value of 42%. For the Micro surface, the values ranged from 20 to 55%, with an average value of 35%. On the 7th day of culture (D7), the differences between the cellular coverage values for Nano and Micro surfaces increased and were in the range of 60–85% and 20–65%, respectively. The average endothelial cell coverage was 73% for Nano and was significantly larger than for Micro samples (43%, p < 0.05).

Cell viability analysis (Fig. 6C) confirmed the microscopic observations. At each time point, the fluorescence value was higher for Nano than Micro surfaces.

#### Magnetic seeding on cylindrical material – 3D model

**Fig. 7:**
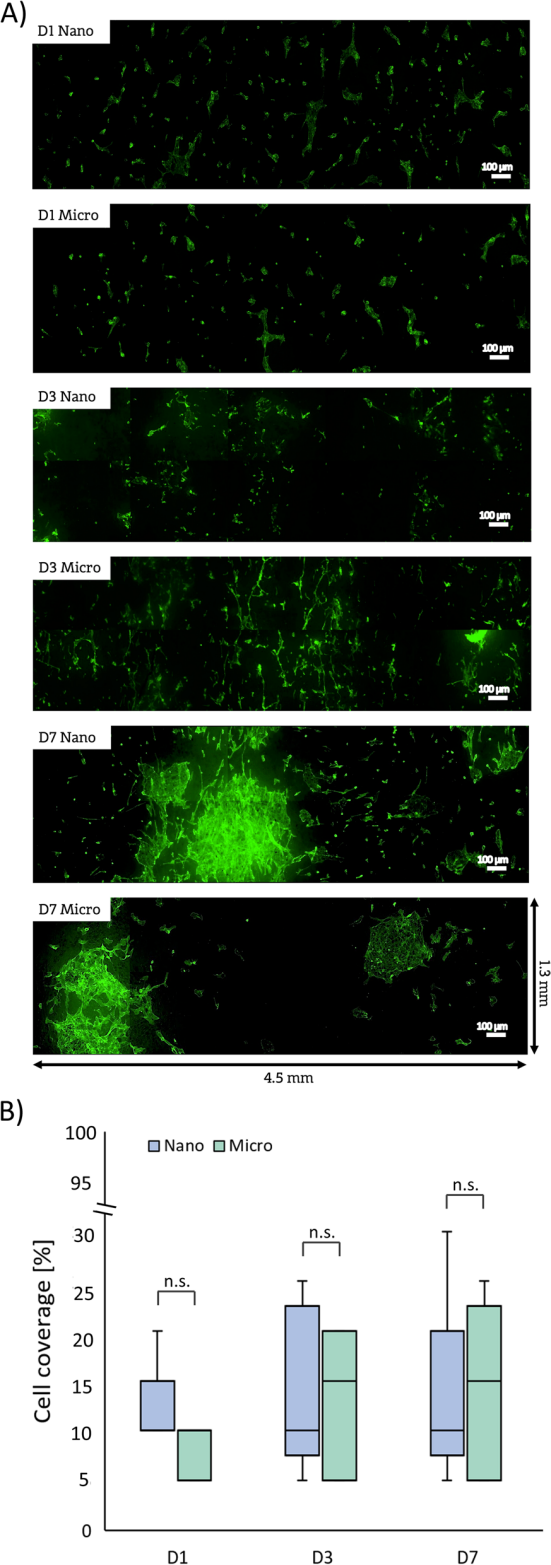
Cell culture with cylindrical materials: (**A**) HUVECs growth and (**B**) cell coverage after 1, 3, and 7 days of culture (*n* = 5)

The results of cell seeding in the 3D cylindrical scaffolds are shown in Fig. 7. Both, morphology of the ECs (Fig. 7A) and the cell coverage values (Fig. 7B) indicate that no significant differences were observed between Nano and Micro prostheses. It is worth emphasizing that cell growth was highly heterogeneous, especially in the later days of culture. On the 7th day of culture, both types of prostheses showed areas of cells forming monolayer-like spots, but there were also areas without any adherent cells. Generally, in both cases, the cellular coverage values were significantly lower than in the 2D culture, being in the range of 5%—30% for Nano and 5%- 25% for Micro prostheses. There were also large differences in the values ​​obtained between multiplicate experimental samples.

## Discussion

Previous studies on small-diameter vascular grafts have revealed that layered structure of vascular prostheses significantly improves their mechanical properties and better mimics the structure and functions of native blood vessel [30]. Each layer of such prosthesis should fulfill certain requirements to enable vessel multifunctionality including anti-thrombogenic function, inhibition of intimal hyperplasia, reduction of inflammation, and enhancement endothelialization after implantation.

Many research groups have previously developed multilayered prostheses, however, most commonly each layer was produced by a different method and often from different polymers. For instance, Yuan et al. created prostheses in which inner, middle, and outer layers were produced by ink printing, wet spinning, and electrospinning, respectively. The authors claimed that only the combined use of the 3 methods allowed for the production of prostheses with the desired wall thickness and mechanical properties [31]. By combining E-jet 3D printing and electrospinning methods, Huang et al. produced tri-layered prostheses, which exhibited better mechanical properties in comparison to electrospun monolayer grafts [30]. Generally, fibrous constructs are very popular in vascular engineering, due to their 3D structure properties mimicking extracellular matrix. Moreover, they offer the possibility to customize fibrous scaffold surface properties (fiber diameter, fiber alignment, material porosity) during production, depending on the requirements of the selected cell type [32].

In this study, multi-step solution blow spinning of medical grade PU enabled us to produce two types of layered fibrous vascular prostheses that differ in their luminal surface morphology. Both types of prostheses were made of three main layers. The outer layer, identical to the Micro and Nano type, was made of microfibers with an average diameter of 1 µm. This layer was designed to support the development of SMCs that build the walls of native blood vessels. In our previous studies, the growth of SMCs on fibers with average diameters in the range of 200, 500, and 900 µm was analyzed [27], showing that SMC growth on fibrous scaffolds with fiber diameters of ~ 1 µm is improved in comparison to smaller diameters. In addition, other studies suggested that not only the size but also the orientation of fibers supports the process of SMC attachment and growth [33, 34]. Based on those results, the outer surfaces of both types of prostheses were designed to contain homogeneous, circumferentially oriented microfibers with average fiber diameter of ~ 1 µm.

A similar diameter was selected to produce a middle layer of the prostheses, composed of loose, non-aligned microfibers. The main task of this ~ 560 µm thick intermediate layer was to ensure the appropriate mechanical properties, which are an important factor determining the success of grafts upon implantation. In vivo, blood vessels are constantly exposed to pulsatile pressure and undergo constant deformation [35], so the prostheses should be produced from highly elastic materials. Furthermore, differences in mechanical properties between the implanted prosthesis and the native vessel can lead to aneurysm formation [36] or anastomotic intimal hyperplasia [37]. Mechanical properties of small-diameter vascular grafts should therefore be similar to properties of native vessels, which they are intended to replace (e.g. coronary artery), or to the commonly used autografts, such as saphenous vein, with longitudinal elastic modulus about 24 MPa and ultimate tensile stress about 6 MPa, or internal thoracic (mammary) artery, with longitudinal elastic modulus about 17 MPa and ultimate stress about 4 MPa [38]. In this study, maximum load values of about 60 N and ultimate tensile stress values of about 10 MPa confirmed high mechanical strength of the produced scaffolds. The prostheses had Young’s modulus values of about 2.5 MPa, which proves their elasticity. It must be noted that Young's modulus of electrospun polyurethane prostheses strongly depends on the type of polymer used, e.g. values reported for Cardiomat were below 1 MPa [39] and for Tecothane around 6 MPa [40]. Grasl et al. reported electrospun Pellethane prostheses with an average fiber diameter of about 900 nm and axial Young’s modulus reaching 10 MPa [41].

The mechanical properties of prostheses change with the change in the average diameter of the fibers that build their walls [42]. As the morphology and thickness of the intermediate layer were the same for Nano and Micro prostheses, it was expected that their mechanical properties will be comparable. The additional nanofiber layer in the Nano-type prostheses was only about 10 µm thick and had therefore no significant effect on the mechanical properties of the entire prosthesis.

Generally, porous structures that mimic extracellular matrix provide a suitable microenvironment for cell growth and tissue regeneration. However, in the case of vascular grafts, they pose a risk of leakage [43]. To overcome this problem, a low-porosity, impermeable compact layer made of densely arranged microfibers was added during fabrication by changing the nozzle-collector working distance, so that the resulting final porosity of the wall was about 40%. This approach allowed us to effectively prevent the leakage as demonstrated in the closed flow system perfusion tests.

The main goal of this study was to evaluate whether the change in the morphology of the internal surface of the prosthesis, without the change in its mechanical properties, has a significant impact on the adhesion of platelets and ECs. Previous studies indicated that cell growth on unmodified polymers including PU is at most moderate and that chemical modifications of the surface, e.g. by introducing peptides, are necessary in order to create a stable layer of the endothelium [44, 45]. On the other hand, many studies reported a strong influence of fiber diameter on cell adhesion. Taking this into account, we analyzed the adhesion of platelets and ECs on the internal surfaces of the prostheses. The adhesion of blood platelets to the inner surface of the prosthesis is an undesirable phenomenon that may lead to the formation of a clot and thrombotic occlusion of the prosthesis lumen. It is also known that the process of platelet adhesion can be influenced by the physical properties of the surface, i.e. roughness [46], topography [47], sub-micron texturing [48]. Studies of platelet adhesion to a solvent-cast film coated with electrospun nanofibers made from poly[acrylonitrile-*co*-(*N*-vinyl-2-pyrrolidone)] (PANCNVP) [49] demonstrated that while the platelets did not adhere to the surface of the film, they did adhere to the surface of the nanofibers. In the micro range, however, Milleret et al. reported that electrospun PU scaffolds with smaller fiber diameters (< 1 μm) reduce platelet adhesion [50]. Authors stated ﻿that not only size of the surface features (e.g. fibers), but also differences in roughness are very likely responsible for the differential platelet adhesion. Our study showed that changing the morphology of the internal surface of the prostheses within the range reported here had a negligible effect on the total percentage of platelet-covered surface. Interestingly, however, the change in luminal surface morphology did influence the morphology of the adherent platelets. Nano-type surface promoted the strong flattening of platelets and their aggregates, while on Micro-type surfaces mostly spherical clusters were formed. Such a difference in the morphology of the adherent platelets may affect the level of their activation and, as a result, the probability of thrombosis. In the context of thrombogenicity, it is worth emphasizing that platelet adhesion to our PU prostheses was overall comparable to the PTFE, which is considered a low-thrombogenic material. The ChronoFlex PU used in this study is also characterized by low platelet adhesiveness and has been successfully used in the production of artificial heart, among others.

One of the key aspects of  successful small-diameter vascular grafting is a rapid endothelialization of prostheses [7]. Endothelial monolayer lining the inner surface of arteries, veins and capillaries constitutes a barrier between blood and tissues [51]. Furthermore, vascular endothelium controls and regulates blood flow. Also, an intact and tight endothelium prevents platelet activation, adhesion, and aggregation. This helps to maintain the patency of vascular graft after implantation [52]. In this study, we hypothesized that changing the prostheses’ luminal surface morphology by introducing layer of nanofibers would enhance the EC attachment and ability to form monolayer. Similar effect was previously reported by Chung et al. who increased roughness of the smooth PU films by grafting PU chains with different molecular weights and chain lengths, showing that increased nanoscale surface roughness enhances the adhesion and growth of ECs [53]. Furthermore, studies of endothelial cell growth on the surface of PLC/collagen fibers with diameters of 0.27, 1, 2.39, 4.45 µm showed that cells grown on 0.27 µm fibers formed strong focal adhesion, whereas cells grown on 2.39 and 4.45 µm fibers presented a spindle-shaped morphology with very few focal adhesion points [42]. In our study, the process of cell colonization in 2D (flat samples, cell seeding by sedimentation) was faster on Nano-type surfaces. The difference in the percentage of cell-covered area between the Micro and Nano prostheses was particularly evident in the later days of the culture. After 7 days of culture, the cellular coverage on the Nano surfaces was in the range of 60–85% and the cell growth was relatively uniform on the entire analyzed surface. On Micro surfaces, the cell coverage values were not only lower, but the cell growth was also patchy and non-uniform. This is certainly related to the greater heterogeneity of the surface morphology of the Micro type, which likely translates into non-uniform cell growth. To investigate whether nanofibrous surface morphology promotes endothelialization of 3D constructs, we employed the magnetic cell seeding method, which was successfully used to populate other types of cylindrical biomaterials [29, 54]. However, the obtained cell coverage values were overall lower than in 2D samples (30% after 7 days of culture) and did not significantly differ between both Nano and Micro prostheses. This trend was observed for both types of materials, for all observation time points. The mechanisms of this effect are unclear thus far but may be related to the differences in the seeding process between 2 and 3D samples. In the case of the 2D model, the cell suspension was applied to the upper surface of the flat samples placed in CellCrown inserts and incubated for 24 h, allowing the cells to sediment onto the material under the influence of gravity. In the 3D model, cylindrical samples placed in a vertical position in cell culture tubes were filled with cell suspension and exposed to magnetic field for 15 min. Consequently, the period when the cells had a chance to adhere to the luminal, cylindrical surface of the prosthesis was much shorter in case of 3D samples that were subsequently placed in the incubator and remained in a vertical position during the whole culture time. Thus, the cells that did not effectively attach to the luminal surface during the 15 min exposure to the magnetic field would have fallen to the bottom of the culture tube. Expectedly, gravity did not work in favor of cell adhesion in vertically placed scaffolds leading to relatively poor attachment of endothelial cells to the cylindrical wall of 3D model and explaining the differences in endothelial coverage between 2 and 3D samples.

In summary, multilayered cylindrical prostheses produced from medical-grade PU by solution blow spinning method, represent a promising alternative to autologous vessels or synthetic polymers. While differing in luminal surface morphology, the designed prostheses showed a high elasticity, good mechanical strength, and a platelet adhesion level comparable to PTFE. Changing the luminal surface morphology by adding a nanofibrous layer significantly improved endothelialization of the flat samples. However, this morphological enhancement was not strong enough to show a significant effect during colonization of the entire cylindrical prostheses, which is a more demanding process. Thus, in order to achieve successful cell colonization of 3D cylindrical prostheses, it will be necessary to introduce additional chemical modifications of their surface (such as introduction of bioactive endothelial cell-selective adhesive molecules, e.g. REDV, IKAV) to overcome current limitations and improve endothelialization efficacy.

## Conclusions

This study aimed to develop non-thrombogenic small-diameter vascular grafts with appropriate mechanical properties and to evaluate the influence of luminal surface morphology on scaffold hemocompatibility and endothelial cell attachment. Collectively, our data demonstrate that multistep solution blow spinning method allows to produce cylindrical structures with layers of tailorable thickness and porosity, whose mechanical properties conform to small-diameter vascular grafts. The developed prostheses did not cause hemolysis in contact with blood and there was no significant difference in the percentage of platelet-covered area for Nano and Micro surfaces. Nanofibrous surfaces promoted stronger adhesion of platelets and their aggregates, resulting in the presence of flattened structures. On the contrary, Micro surfaces were characterized by the presence of spherical aggregates, which indicates their weaker adhesion. This variation in surface-adhered platelets might indicate differences in their activation level.

Endothelial coverage after 1, 3, and 7 days of 2D culture was higher on Nano prostheses. However, this effect was not seen in 3D culture, where cylindrical prostheses were colonized using magnetic seeding method. Taken together, the produced scaffolds meet the material and mechanical requirements for vascular prostheses, but their biological properties must be further improved to enhance endothelialization efficiency.

## Supplementary Information

**Additional file 1: Figure. **Cross-sectional SEM images of Nano and Micro prostheses after static (7 and 14 days) and dynamic (1h) delamination test.

## Data Availability

The datasets generated during the study are available from the corresponding author upon reasonable request.

## Abbreviations

ECs: Endothelial cells
EtOH: Ethanol
HMDS: Hexamethyldisilazane
HUVECs: Human umbilical vein endothelial cells
PBS: Phosphate buffer saline
PET: Polyethylene terephthalate
PRP: Platelet-rich plasma
PTFE: Polytetrafluoroethylene
PUs: Polyurethanes
SBS: Solution blow spinning
SEM: Scanning electron microscopy
SMCs: Smooth muscle cells
