# Supplementary figures and images for: Multilayered blow-spun vascular prostheses with luminal surfaces in Nano/Micro range: the influence on endothelial cell and platelet adhesion
Source: J Biol Eng. 2023 Mar 13;17:20. doi: 10.1186/s13036-023-00337-9 (PMC10012602; doi:10.1186/s13036-023-00337-9)

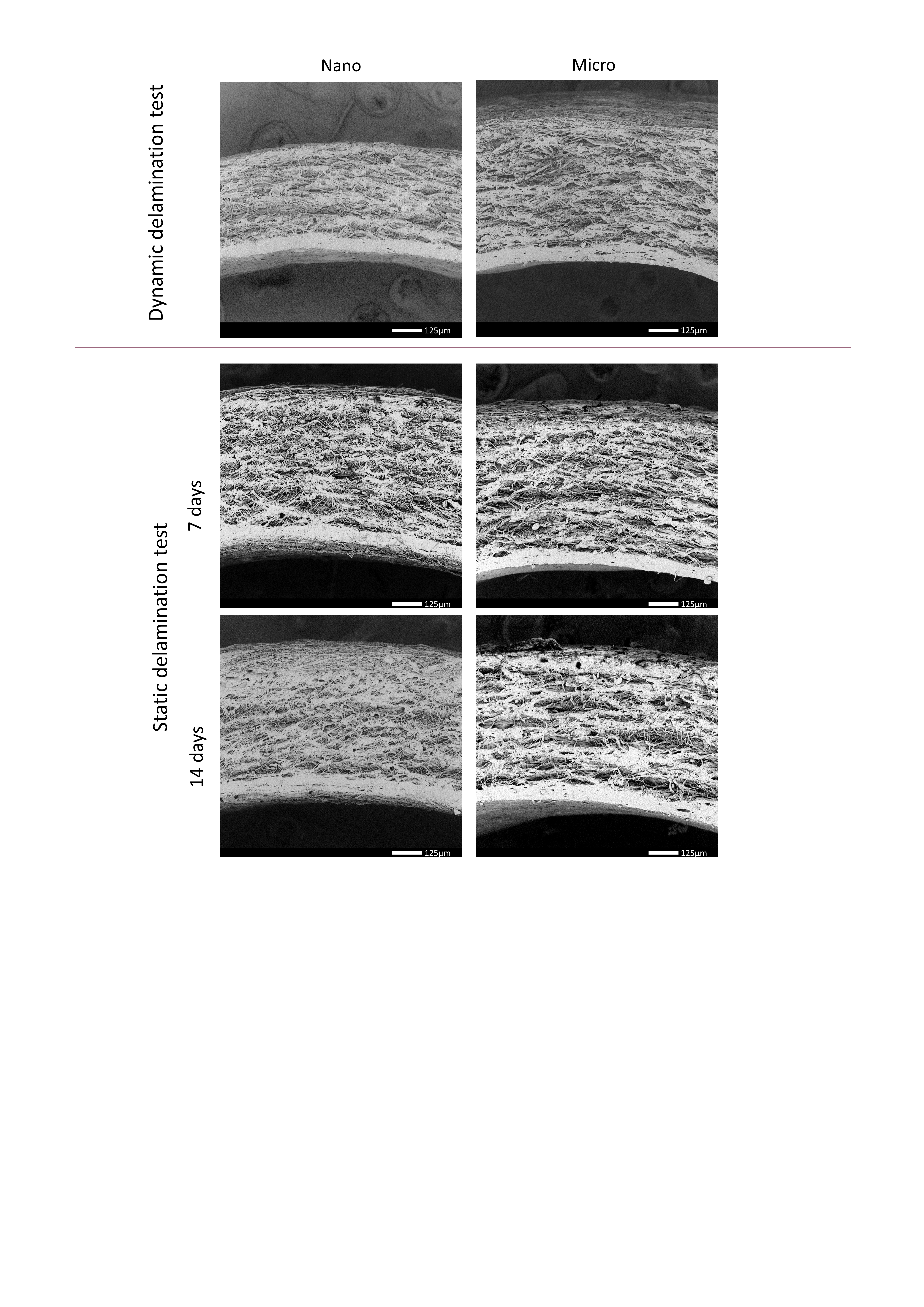

Supplement: Supplementary file 1 — Additional file 1: Figure. Cross-sectional SEM images of Nano and Micro prostheses after static (7 and 14 days) and dynamic (1h) delamination test. [file 13036_2023_337_MOESM1_ESM.tif]
